# Nanoemulsified Butenafine for Enhanced Performance against Experimental Cutaneous Leishmaniasis

**DOI:** 10.1155/2021/8828750

**Published:** 2021-03-31

**Authors:** Adriana Bezerra-Souza, Jéssica A. Jesus, Márcia D. Laurenti, Aikaterini Lalatsa, Dolores R. Serrano, Luiz Felipe D. Passero

**Affiliations:** ^1^Laboratory of Pathology of Infectious Diseases (LIM-50), Medical School, University of São Paulo, Avenida Dr. Arnaldo 455, 01246903 Cerqueira César, SP, Brazil; ^2^Biomaterials, Bio-engineering and Nanomedicines (BioN) Laboratory, Institute of Biomedical and Biomolecular Sciences, School of Pharmacy and Biomedical Sciences, University of Portsmouth, White Swan Road, Portsmouth PO1 2DT, UK; ^3^Department of Pharmaceutics and Food Technology and Instituto Universitario de Farmacia Industrial (IUFI), School of Pharmacy, Complutense University, Avenida Complutense, 28040 Madrid, Spain; ^4^Institute of Biosciences, São Paulo State University (UNESP), São Vicente, Praça Infante Dom Henrique, s/n, 11330-900 São Vicente, SP, Brazil; ^5^São Paulo State University (UNESP), Institute for Advanced Studies of Ocean, São Vicente, Av. João Francisco Bensdorp, 1178, 11350-011 São Vicente, SP, Brazil

## Abstract

The production of ergosterol lipid involves the activity of different enzymes and is a crucial event for the *Leishmania* membrane homeostasis. Such enzymes can be blocked by azoles and allylamines drugs, such as the antifungal butenafine chloride. This drug was active on parasites that cause cutaneous and visceral leishmaniasis. Based on the leishmanicidal activity of butenafine chloride and considering the absence of reports about the therapeutic potential of this drug in cutaneous leishmaniasis, the present work is aimed at analyzing the efficacy of butenafine formulated in two different topical delivery systems, the self-nanoemulsifying drug delivery systems (BUT-SNEDDS) and in a SNEDDS-based nanogel (BUT-SNEDDS gel) as well as in the free form in experimental cutaneous leishmaniasis. Physical studies showed that both formulations were below 300 nm with low polydispersity (<0.5) and good colloidal stability (around -25 mV). Increased steady-state flux was reported for nanoenabled butenafine formulations with reduced lag time in Franz cell diffusion assays across Strat-M membranes. No toxic or inflammatory reactions were detected in animals treated with BUT-SNEDDS, BUT-SNEDDS gel, or butenafine. Animals topically treated with butenafine (free or nanoformulated) showed small dermal lesions and low tissue parasitism. Furthermore, BUT-SNEDD gel and butenafine presented similar efficacy than the standard drug Glucantime given by the intralesional route. Increased levels of IFN-*γ* were observed in animals treated with BUT-SNEDDS gel or butenafine. Based on these data, the antifungal drug butenafine chloride can be considered an interesting repurposed drug for the treatment of cutaneous leishmaniasis.

## 1. Introduction

Leishmaniasis is an infectious disease caused by protozoans from the Trypanosomatidae family, Kinetoplastida order, and *Leishmania* genus, that affect humans, wild and domestic animals, and invertebrates belonging to the *Lutzomyia* and *Phlebotomus* genuses [1, 2]. Leishmaniasis is considered a complex of diseases with important clinical-immunological spectrum and epidemiological diversity. Depending on the infecting species and the intrinsic features of the host, cutaneous or visceral leishmaniasis can be clinically characterized. The cutaneous form of leishmaniasis is caused by different *Leishmania* species; therefore, a spectrum of clinical signs can be found, ranging from a single localized skin lesion, that can heal spontaneously, to multiple ulcerated or nonulcerated lesions in the skin and/or mucosa; these types of lesions frequently require a more complex treatment [3]. In spite of that, the treatment of all clinical forms of leishmaniasis is based on few therapeutic alternatives, such as pentavalent antimonials and amphotericin B [4].

Pentavalent antimonials remain the first choice of treatment for all clinical forms leishmaniasis, mainly in Latin America [5]. Additionally, pentavalent antimonials induce significant side effects such as gastrointestinal intolerance and cardiotoxicity, resulting in low patient compliance and termination of therapy prior to achieving therapeutic outcomes [6]. In some geographic areas, such as in India, drug-resistant parasites have been frequently detected [7]. In such situations, amphotericin B is used as the second-line drug. Amphotericin B is effective in treating leishmaniasis [8], but it interacts with the host cell membrane inducing mild to severe adverse effects in patients, including fever and renal and gastrointestinal toxicities [9, 10]. Moreover, amphotericin B-resistant parasites have been isolated [11]. To mitigate toxicity of amphotericin B micellar formulation, liposomal formulations of amphotericin B are clinically indicated [12], but their use is limited in developing countries due to high cost and temperature instability [12, 13]. An amphotericin B cream (3% *w*/*w*, Anfoleish) is currently under clinical trials, but preliminary results have shown variable efficacy in patients with CL as a result of limited skin permeability [14], while a range of adverse effects such as itching, redness, peeling dryness, and irritation of the skin were observed in patients [15]. Miltefosine, the only orally bioavailable licensed treatment for leishmaniasis, has shown different levels of efficacy [16]. Paromomycin, only available licensed formulation, has shown poor efficacy in treating post-kala-azar dermal leishmaniasis in India; however in the New World, it shows variable efficacy in cutaneous leishmaniasis [17, 18]. Altogether, the prevalence of the disease with distinct outcomes, the ineffectiveness, and toxicity of the available drugs emphasizes the need for more active and less toxic treatments based on natural or synthetic molecules [19–21].

The sterol biosynthesis pathway is shared by fungi and *Leishmania* sp. [22, 23]. Molecules generated in this biochemical pathway, such as ergosterol and other 24-methyl sterols, are important for the maintenance of the cell membrane homeostasis. In fact, studies already showed that antifungal drugs are active on *Leishmania* parasites, and these drugs can be selective toward parasites, since host cells do not produce ergosterol, and depending on the drug, the impact towards the homeostasis of the host can be absent or tolerable [24, 25]. The class of the antifungal azoles such as ketoconazole, fenticonazole and tioconazole, that were previously shown to inhibit the C14*α*-demethylase enzyme, was able to eliminate promastigote and amastigote of *Leishmania* sp. *in vitro* and *in vivo* [26, 27]. Additionally, squalene epoxidase enzyme, that converts squalene to lanosterol, an important precursor of ergosterol, has also been successfully inhibited by antifungal drugs belonging to the allylamine class [28, 29]. The most studied allylamine drug so far is terbinafine that was active on promastigote and amastigote forms of *Leishmania* sp. [30, 31]. Additionally, patients with CL treated with terbinafine by the oral route showed partial to full recovery [32], while cutaneous lesions of patients treated with topical terbinafine (32.25–75.5 mg/day depending on the size of the skin lesion) plus Glucantime (20 mg/kg by intramuscular route) during 20 days showed faster improvement in comparison to patients treated with placebo ointment [33].

Besides terbinafine, other antifungal drugs that target squalene epoxidase enzyme impacted *Leishmania* sp. survival. Butenafine hydrochloride and tolnaftate drugs, that are traditionally indicated for the topical treatment of superficial mycosis, were active on promastigote and amastigote forms of *L. (L.) amazonensis*, *L. (V.) braziliensis*, and *L. (L.) infantum* [34, 35], and by morphological and/or physiological studies, the lipids from parasites were affected during the *in vitro* treatments. These and other studies highlight that squalene epoxidase enzyme is an attractive target to be inhibited aiming at impairing the parasite viability.

In spite of elegant works on drug repurposing in leishmaniasis, few reports provided *in vivo* validation of drug candidates. To the best of our knowledge, this is the first study to demonstrate the *in vivo* efficacy of butenafine in cutaneous leishmaniasis. Here, we present a topical butenafine formulation that involves loading butenafine in self-nanoemulsifying drug delivery systems (SNEDDS) and SNEDDS-enabled hydrogels in an attempt to improve butenafine permeation across the skin and localize effective concentrations butenafine within the dermis, increasing the efficacy of butenafine in American cutaneous leishmaniasis.

## 2. Material and Methods

### 2.1. Materials

Butenafine hydrochloride (>98%, HPLC) was obtained from Kemprotec Ltd. (Smailthorn, Middleton-in-Lonsdale, Cumbria, UK). Labrasol (caprylocaproyl macrogol-8 glycerides), Transcutol P (diethylene glycol monoethyl ether), and Capryol 90 (propylene glycol monocaprylate) were a gift from Gattefosse (Alpha Chemicals, Berkshire, UK). Carbopol 940 and all other chemicals were purchased from Fisher Scientific UK (Loughborough, UK).

### 2.2. Preparation of Butenafine Nanoformulations

BUT-SNEDDS (2.125% *w*/*w*) were prepared by dispersing BUT (0.0425 g) within an isotropic mixture of Labrasol (0.6 g), Capryol 90 (0.2 g), and Transcutol P (1.2 g), respectively [36, 37]. The ratio of oil : surfactant and solvent was optimized in terms of particle size using tertiary diagrams, and choice of surfactants and solvents was based on solubility studies [36, 37]. The BUT-SNEDDS were magnetically stirred for 15 minutes and left under stirring in a water bath (50 rpm, Kotterman D1365, Hanigsen, Germany) at 37°C overnight for 16 hours [20]. Blank SNEDDS were produced using the same methodology but without adding BUT.

To prepare BUT-SNEDDS gel (0.70% *w*/*w*), Carbopol 940 (1 g) was added in deionized water (25 mL) and left to swell overnight. The pH of the swollen gel (10 g) was then adjusted to pH 6.5 by addition of sodium hydroxide (~0.78 mL, 5 M). Neutralised Carbopol 940 gel (10 g) and BUT-SNEDDS (2.125% *w*/*w*, 5 g) (final pH: 6.5 ± 0.1, Accumet AB200 pH meter, Fisher Scientific, Loughborough, UK) were mixed to obtain BUT-SNEDDS gel.

### 2.3. Characterization of Prepared SNEDDS and SNEDDS Gel in terms of Particle Size and Colloidal Stability

Blank and butenafine-loaded SNEDDS and SNEDDS gels were diluted with deionized water (pH 6.5 ± 0.1) (5 mg in 30 mL of water and 16.8 mg in 1.5 mL of water, respectively). SNEDDS samples were vortexed and left to stand for 15 minutes prior to analysis. Gels were diluted and centrifuged (5,000 rpm, 5 minutes, SciSpin, Micro Centrifuge, Shropshire, UK) to remove carbomer, which is insoluble in water, and the supernatant was left to stand for 15 minutes prior to analysis. Particle size and zeta potential were measured as previously described [13, 20, 36, 38] using a Nano-ZS Zetasizer (Malvern Instruments, Worcestershire, UK). The data were analyzed using the Contin method of data analysis [36]. The accuracy of the instrument was assessed periodically using a drop of latex beads (polystyrene, mean size 0.1 *μ*m) in 50 mM sodium chloride (dispersed phase). All measurements (*n* = 13) were performed in triplicate, and results presented as the mean ± SD were reported.

Zeta potential (Malvern Nano-Zs, Malvern Instruments, UK) was measured for the diluted formulations using the Doppler electrophoresis technique. Analysis of the Doppler shift (Fourier transformed) was done by using mixed-mode measurement phase analysis light scattering (M3-Pals). The viscosity of the sample was hypothesized to be the viscosity of water at 25°C. All measurements were performed in triplicate, and results presented as the mean ± SD were reported [20].

### 2.4. Franz Cell Diffusion Studies

Franz cells (of 12 mL capacity) were mounted with a semisolid Teflon holder with a diffusional area of 1.327 cm^2^. Compartments were rinsed with deionized water and methanol, and a stirrer bar (3 × 6 mm) was placed inside. To ensure sink conditions, the receptor compartment was filled with a mixture of 0.1% *v*/*v* trimethylamine buffer (adjusted to pH 5.00 ± 0.1 using 1 M hydrochloric acid and 1 M sodium hydroxide when needed) and methanol (9 : 1 *v*/*v*), preheated to 37°C. Strat-M membranes for transdermal diffuse testing (Millipore) were mounted to adequately cover the receptor chambers. The donor compartment and the receptor compartment were tightly sealed using a thin layer of KORASILON Paste silicone grease (Mittelviskos Kurt Obermeier GmbH & Co. KG) and Parafilm™ prior to being clamped together. The donor chamber was filled with 0.1% trimethylamine buffer (0.4 mL) and covered with Parafilm™ prior to being placed in a water bath at 37°C (RCT basic, IKA® England Ltd., Oxford, UK). After 0.5 h, the buffer was removed from the receptor chamber and was collected for analysis. The receptor chamber was refilled with fresh trimethylamine buffer and methanol mixture prewarmed to 37°C. The trimethylamine buffer in the donor chamber was removed, and the formulations (BUT SNEDDS 1% or BUT SNEDDS gel 1%; 0.4 g) or butenafine solubilized in PBS plus 1% DMSO (10 mg/mL; 0.4 mL/chamber) was added to the donor chamber ensuring it was in contact with the Strat-M membranes. Samples (0.3 mL) were withdrawn at predetermined times (5 min, 10 min, 15 min, 30 min, 60 min, 120 min, 180 min, 240 min, 360 min, and 480 min) from the receptor chamber using a 1 mL syringe with a 21 g needle (38 mm in length), and samples were analyzed by HPLC as described below. The receptor chamber was immediately replenished with prewarmed trimethylamine buffer and methanol mixture (0.3 mL).

Collected samples were analyzed by HPLC which was equipped with a Jasco PU-1580 pump, a Jasco AS-2050 Plus autosampler, and a Jasco UV-1575 UV-visible detector. Integration of the peaks was performed with the program Borwin 1.5 for PC (JMBS Developments). A Phenomenex LiChrosorb C18 reverse phase HPLC column (250 × 2.6 mm, 5 *μ*m, 100 Å) was used for analysis. An isocratic elution was used with a mobile phase consisting of freshly prepared and 0.45 *μ*m nylon filtered 0.1% *v*/*v* trimethylamine buffer (adjusted to pH 5.00 ± 0.1 using 1 M hydrochloric acid and 1 M sodium hydroxide when needed) and HPLC grade acetonitrile (25 : 75 *v*/*v*). The flow rate was set at 1.2 mL/min, and the injection volume was 40 *μ*L. Detection was carried at 282 nm and a linear calibration curve was achieved between 0.1 and 100 *μ*g mL^−1^ (*R*^2^ > 0.999).

Regression analysis was used to calculate the slopes and intercepts of the linear portion of each graph. The steady-state flux (JSS), permeability coefficient (*P*), the diffusion coefficient, and the lag time were estimated as previously described in Lalatsa et al. [36]. Each formulation was tested at least in triplicate.

### 2.5. Animals

Six- to eight-week-old female BALB/c mice were obtained from Medical School of São Paulo University. This study was carried out in strict accordance with the recommendations in the *Guide for the Care and Use of Laboratory Animals* of the Brazilian National Council of Animal Experimentation (http://www.cobea.org.br). The protocol was approved by the Committee on the Ethics of Animal Experiments of the Institutional Animal Care and Use Committee at the Medical School of São Paulo University (CEP 322/12). For all experimental procedures, mice were anaesthetized or euthanized with thiopental (50 and 150 mg/kg, respectively).

### 2.6. Histological Changes in the Skin of Healthy BALB Mice Treated with Butenafine-Containing Nanoformulations

Thirty-five female BALB/c mice were divided into seven groups: group 1 was treated topically with SNEDDS (containing 10 mg of butenafine); group 2 was treated topically with BUT-SNEDDS gel (containing 10 mg of butenafine), group 3 was treated topically with butenafine solubilized in DMSO (10 mg of butenafine), and group 4 was injected intralesionally with 100 mg/kg of Glucantime. Groups 5 and 6 were topically treated with blank SNEDDS or blank SNEDDS gels, respectively. Group 7 was topically treated with vehicle solution (PBS plus 1% DMSO). Animals were treated once a day for 15 days. Animals were physically examined weekly. Forty-eight hours after the last application, animals were euthanized with thiopental. Skin fragments were collected, fixed in formalin, and stained with hematoxylin and eosin to analyze histological changes.

### 2.7. Parasites

*L. (L.) amazonensis* parasite (MHOM/BR/73/M2269) was kindly provided by Prof. Dr. Fernando T. Silveira from the criobank of “Leishmaniasis Laboratory Prof. Dr. Ralph Laison”, Department of Parasitology, Evandro Chagas Institute, Ministry of Health, Belém, Pará, Brazil. The parasite was identified using monoclonal antibodies and isoenzyme electrophoretic profiles at the Leishmaniasis Laboratory of the Evandro Chagas Institute (Belém, Pará State, Brazil). This parasite was grown in Roswell Park Memorial Institute-1640 medium—RPMI 1640 (Gibco^®^, Life Technologies, Carlsbad, CA, USA), supplemented with 10% heat-inactivated fetal bovine serum, 10 *μ*g/mL of gentamicin, and 1,000 U/mL of penicillin (R10) at 25°C. Promastigote forms in the stationary phase were used.

### 2.8. Infection and Experimental Treatment

Thirty-five male BALB/c mice were subcutaneously infected in the base of tail with 10^6^ promastigote forms of *L. (L.) amazonensis*, and five BALB/c mice received only sodium chloride 0.9% (*w*/*v*) under the same route (healthy group). Four weeks after infection, *L. (L.) amazonensis*-infected BALB/c mice were divided into seven groups: group 1 (G1) was constituted by infected animals that received only vehicle solution (PBS plus 1% DMSO); groups 2 (G2) and 3 (G3) were treated with blank SNEDDS or blank SNEDDS gels, respectively; group 4 (G4) was treated with BUT-SNEDDS (containing 10 mg of butenafine); group 5 (G5) was treated topically with BUT-SNEDDS gel (containing 10 mg of butenafine), group 6 (G6) was treated topically with butenafine (10 mg of butenafine) solubilized in PBS plus 1% of DMSO, and group 7 (G7) was injected intralesionally with 100 mg/kg of Glucantime. Groups 1 to 6 were treated topically with butenafine-containing formulations, blank formulations, butenafine, or vehicle solution, while G7 was injected intralesionally. Group 8 was constituted by noninfected, nontreated animals. Animals were treated for 15 consecutive days once daily. The physical conditions of the animals were monitored once a week. Two weeks after the last application, animals were euthanized with thiopental. Skin fragments were collected, fixed in formalin, and stained with hematoxylin and eosin to analyze histological changes. There was no dead prior to the endpoint. In all experiments, parasites were in stationary phase of growth, and never excessed three passages *in vitro*.

### 2.9. Clinical Course of Lesion Development and Determination of Parasite Burden in the Skin of Infected and Treated Animals

The clinical course of lesion development was evaluated weekly by recording the average diameter of the tail measured as the mean of tail base diameters in horizontal and vertical directions using a caliper. The parasite load in the skin was determined using the quantitative limiting dilution assay, as previously described [39]. Briefly, a skin fragment from the base of tail was aseptically excised, weighed, and homogenized in Schneider's medium. The skin suspensions were subjected to 12 serial dilutions with four replicate wells. The number of viable parasites was determined based on the highest dilution that promastigotes could be grown after 10 days of incubation at 25°C.

### 2.10. Cytokine Production Studies

The subiliac and popliteal lymph nodes from the different groups were aseptically collected and macerated in R10 medium, and the number of cells estimated under trypan blue exclusion dye. Cells were adjusted at 5 × 10^5^ cells/well and stimulated with 5.0 *μ*g of whole antigen of *L. (L.) amazonensis* or 1.0 *μ*g of concanavalin A, as a positive control; negative controls were cultivated only with R10 medium during 72 h in a humidified incubator, 37°C, 5% CO_2_. Following this experimental time, supernatants were collected, and the amounts of IL-4 and IFN-*γ* (BD, Franklin Lakes, NJ, USA) were quantified by sandwich enzyme-linked immunosorbent assay (ELISA) in accordance with the manufacturer's recommendations.

### 2.11. Statistical Analysis

The results were expressed as the mean ± standard deviation of three independent experiments, and the nonparametric Mann–Whitney *U* test was used to compare results among groups. Differences were considered statistically significant at 5% significance level (*p* < 0.05). GraphPad Prism 5 (GraphPad Software, Inc., La Jolla, CA, USA) was used to analyze the results.

## 3. Results

### 3.1. Measurement of Particle Size and Zeta Potential of Nanoformulations

Prepared BUT-SNEDDS and BUT-SNEDDS gel illustrated sizes consistently below 300 nm (Table 1) with good colloidal stability. The particle size of BUT-SNEDDS and BUT-SNEDDS gel was similar indicating the ability of nanoparticles forming after dilution of the gels in aqueous environments. Viscosity of the prepared hydrogels was appropriate for skin application avoiding running [36].

### 3.2. Franz Cell Diffusion Studies

In the permeability studies, it was observed that butenafine-containing formulations displayed higher flux rate, permeability, and diffusion coefficients through the Strat membrane in comparison to the butenafine solubilized in DMSO (*p* < 0.05). Additionally, the formulations showed a significant lower lag time compared to free butenafine which can be explained for the slower release of the drug (*p* < 0.05). These data are summarized in Table 2.

### 3.3. Histological Changes in the Skin of Healthy BALB Mice Treated with Butenafine-Containing Nanoformulations

BALB/c mice were treated topically with formulations containing butenafine (10 mg of butenafine), butenafine solubilized in DMSO (10 mg), and blank formulations or intralesionally treated with Glucantime (100 mg/kg) once a day during 15 days. Forty-eight hours later, animals were euthanized and fragments of the skin were collected.

**Figure 1:**
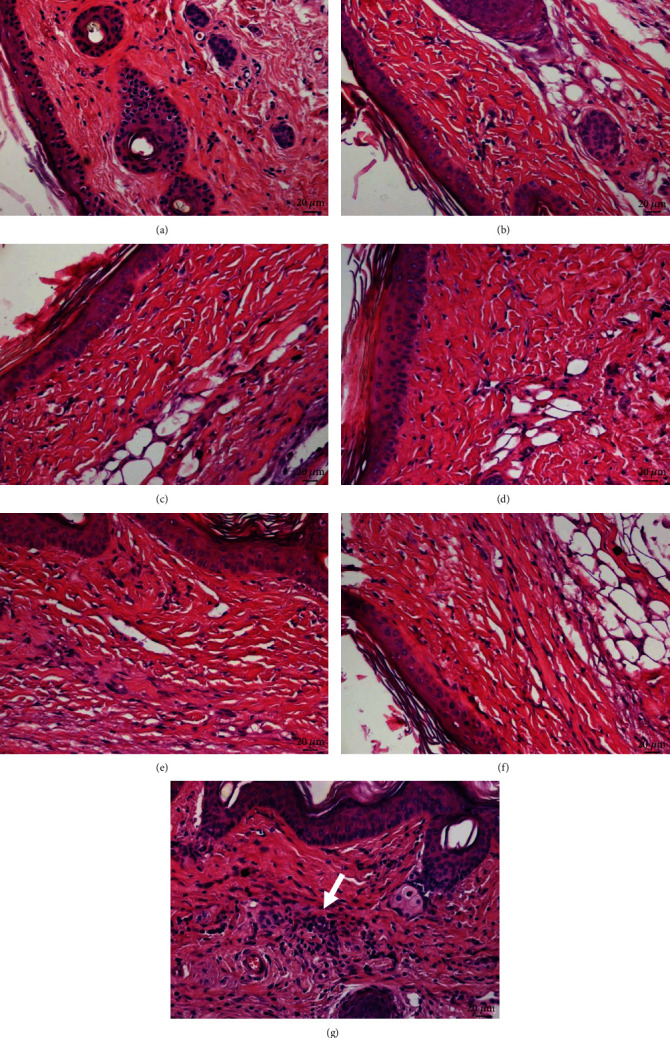
BALB/c mice were treated topically with butenafine chloride formulated in the self-nanoemulsifying drug delivery system (SNEDDS) or in a SNEDDS-based nanogel (SNEDDS gel) containing butenafine (10 mg of butenafine per dose), butenafine solubilized in DMSO (10 mg/dose), and blank formulations of the nanosystems or intralesionally treated with Glucantime (100 mg/kg/dose) once a day during 15 days. Forty-eight hours after the last dose, fragments of the skin from BALB/c mice were collected and analyzed by histology. Histological section of the skin from (a) nontreated animals, (b) blank SNEDDS, (c) blank SNEDDS gel, (d) BUT-SNEDDS, (e) BUT-SNEDDS gel, (f) butenafine, and (g) Glucantime. White arrow shows an area of inflammatory infiltrate.

The histological section of the skin from nontreated BALB/c mice showed no morphological changes in the epidermis and dermis layers (Figure 1(a)). Similarly, skin from animals treated with blank SNEDDS and blank SNEDDS gels (Figures 1(b) and 1(c), respectively), BUT-SNEDDS and BUT-SNEDDS gel (Figures 1(d) and 1(e), respectively), or butenafine solubilized in DMSO (Figure 1(f)) showed normal morphology of the epidermis and dermis; additionally, no signs of inflammation were observed. Animals treated with Glucantime (Figure 1(g)) did not show changes in the epidermis; however, a diffuse inflammatory infiltrate was identified in the dermis, mainly composed of mononuclear cells (Figure 1(g), white arrow).

### 3.4. Clinical Course of the Lesion Development and Determination of Parasite Burden in the Skin of Infected and Treated Animals

**Figure 2:**
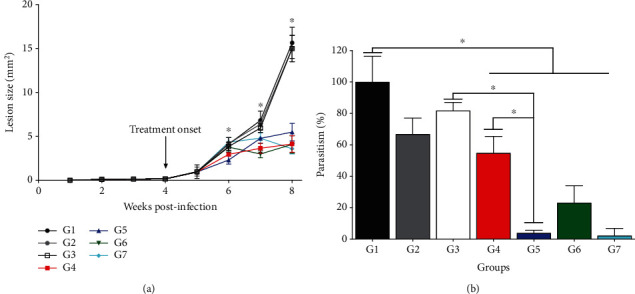
*In vivo* efficacy of butenafine and nanoenabled formulations in experimental cutaneous leishmaniasis. BALB/c mice were infected into the base of the tail with 10^6^ promastigote forms of *L. (L.) amazonensis* in stationary phase of growth. Four weeks after, infected animals were topically treated once daily for 15 days with blank SNEDDS, blank SNEDDS gel, BUT-SNEDDS, BUT-SNEDDS gel, butenafine, and Glucantime. Lesion sizes were analyzed weekly (a), and the skin parasitism from the base of tail, quantified by limiting dilution assay, was analyzed at 8 weeks of postinfection (b). ^∗^*p* < 0.05 indicates statistical significance. G1: infected control; G2 and G3: animals treated with blank SNEDDS or blank SNEDDS gels, respectively; G4: animals treated with BUT-SNEDDS; G5: animals treated BUT-SNEDDS gel; G6: animals treated with butenafine; G7: animals treated with Glucantime.

All the infected control groups [infected nontreated (G1), treated with blank SNEDDS (G2), or blank SNEDDS gel (G3)] showed similar growth of lesions that increased over eight weeks of postinfection (Figure 2(a)). Skin lesions in animals treated with BUT-SNEDDS (G4) and BUT-SNEDDS gel (G5) as well as butenafine (G6) and Glucantime (G7) significantly decreased in size after 6 weeks of postinfection and remained significantly smaller until the end of the experiment (8 weeks) compared to the control groups (*p* < 0.05, Figure 2(a)).

In comparison to the controls, animals treated topically with nanoenabled butenafine formulations or butenafine presented lower tissue parasitism (*p* < 0.05). Additionally, animals treated with Glucantime (G7) by the intralesional route also showed low parasitism in the skin compared to the controls (*p* < 0.05). Furthermore, BUT-SNEDDS gel (G5) was more efficient at decreasing tissue parasitism in infected animals than blank SNEDDS gel (G3). Although efficient at decreasing the lesion size, BUT-SNEDDS (G4) displayed similar ability to decrease parasite load than blank SNEDDS (G2) (*p* > 0.05). Treatment with BUT-SNEDDS gel (G5) and butenafine (G6) demonstrated comparable efficacy to intralesional administration of Glucantime (G7) (*p* > 0.05), as shown in Figure 2(b).

### 3.5. Histological Changes in Infected Animals Treated with Free or Nanoformulated Butenafine

**Figure 3:**
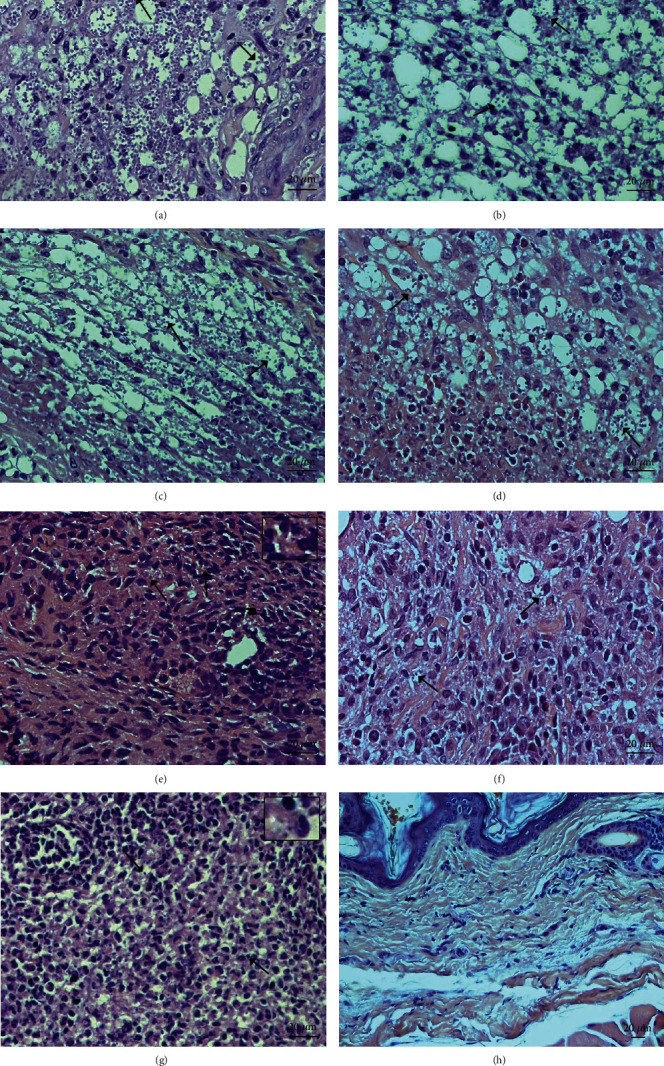
Skin histological section from infected controls: (a) infected, nontreated, (b, c) infected and treated with blank SNEDDS or blank SNEDDS gel, respectively, and animals treated with BUT-SNEDDS or BUT-SNEDDS gel (d, e, respectively), butenafine (f), or Glucantime (g). Skin histological section from healthy animals is shown in (h). Black arrows indicate intracellular amastigote forms. Insets show in details amastigote forms of the skin histological sections from animals treated with butenafine loaded in gel (e) or Glucantime (g).

Histological sections from the skin of infected control animals, i.e., infected nontreated (G1), treated with blank SNEDDS (G2), or with blank SNEDDS gels (G3) as in Figures 3(a)–3(c), respectively, displayed complete disruption of the epidermis and dermis. Macrophages were highly infected with amastigotes in such control groups; additionally, neutrophil and eosinophil immune cells were detected throughout these sections. Histological sections from animals treated with BUT-SNEDDS (G4) displayed lower tissue parasitism compared to the controls (G1, G2, and G3), but mixed inflammatory infiltrate still persisted, with the involvement of mononuclear and polymorphonuclear immune cells (Figure 3(d)). On the other hand, lesions from animals treated topically with BUT-SNEDDS gel (G5) (Figure 3(e)) or intralesionally with Glucantime (G7) (Figure 3(g)) showed inflammatory infiltrates characterized by the presence of lymphocytes and few infected macrophages (inset in the respective figures). In the histological section of the skin from BALB/c mice treated with free butenafine (G6) (Figure 3(f)), inflammatory infiltrate was constituted of mononuclear cells, mainly with the involvement of few polymorphonuclear cells.  Figure 3(h) shows histological section from the skin of healthy BALB/c mice (G8). Black arrows indicate amastigote forms.

### 3.6. Cytokine Production Studies

**Figure 4:**
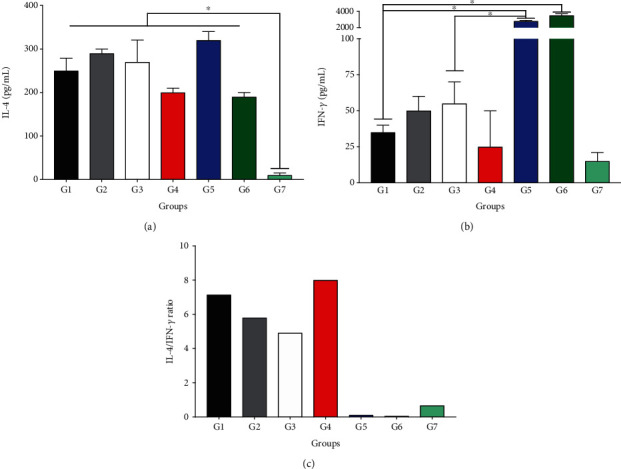
Mononuclear cells from lymph nodes of treated and control BALB/c mice were isolated and cultured by 72 h under specific stimulation with the whole antigen of *L. (L.) amazonensis*, following the levels of IL-4 (a) and IFN-*γ* cytokines (b) which were quantified by ELISA. (c) IL-4/IFN-*γ* ratio. ^∗^*p* < 0.05 indicates statistical significance. G1: infected control; G2 and G3: animals treated with blank SNEDDS or blank SNEDDS gels, respectively; G4: animals treated with BUT-SNEDDS; G5: animals treated with BUT-SNEDDS gel; G6: animals treated with butenafine; G7: animals treated with Glucantime.

Mononuclear cells from animals treated with blanks (G2 and G3), BUT-SNEDDS (G4), SNEDDS gel (G5), and butenafine (G6) produced similar amounts of IL-4 (Figure 4(a)) in comparison to the infected control animals (G1). Cells from animals treated with Glucantime (G7) produced significant low levels of IL-4 in comparison with the infected control group (*p* < 0.05).

In comparison with infected control (G1), mononuclear cells from animals treated with BUT-SNEDDS gel (G5) or butenafine (G6) produced significant high levels of IFN-*γ* (*p* < 0.05). Cells from animals treated with blanks (G2 and G3), BUT-SNEDDS (G4), or Glucantime (G7) did not alter the amounts of IFN-*γ* produced (*p* > 0.05, Figure 4(b)). Animals treated with BUT-SNEDDS gel (G5) produced higher amounts of IFN-*γ* than animals treated with blank SNEDDS gel (G3) (*p* < 0.05).

Furthermore, it was possible to observe that the control groups (G1, G2, and G3) showed an elevated IL-4/IFN-*γ* ratio, demonstrating that these animals from an immunological point of view developed an expressive Th2 immune response, although the G4 group, treated with BUT-SNEEDS, also developed a Th2 response. In contrast, animals treated with BUT-SNEDDS gel (G5), butenafine (G6), and Glucantime (G7) were able to inhibit the production of Th2 immune response, and especially, G5 and G6 upregulated Th1 immune response.

Lymph node cells stimulated with concanavalin A produced high amounts of both cytokines (data not shown), while negative controls (cells cultured with R10 only) did not produce quantifiable levels of both cytokines (data not shown).

## 4. Discussion

The cell membrane physiology of *Leishmania* parasites is dependent on the formation of ergosterol and other 24-alkyl sterols; furthermore, this biochemical route is complex and different leishmanial enzymes take part of this process. Thus, the inhibition of key molecules may disrupt the balance of the cell membrane and induce death in *Leishmania* sp., caused by depletion of ergosterol precursors [40]. Butenafine, an antifungal drug, has been shown to eliminate promastigote and intracellular amastigote forms of *L. (L.) amazonensis* and *L. (V.) braziliensis* selectively, while being able to induce structural changes associated with lipid recycling and programmed cell death in promastigote forms of *L. (L.) amazonensis* [34]. Possibly, the leishmanicidal activity of butenafine relies on the fact that it is able to inhibit squalene epoxidase enzyme, and once inhibited, squalene as well as other intermediated molecule will not be produced, resulting in a deficiency in basic processes required for *Leishmania* sp. survival, such as membrane recycling and cell division [29].

Although butenafine is active on *Leishmania* sp. parasites [41], there are not available clinical formulations to support its topical use for the treatment of cutaneous leishmaniasis. Thus, this study demonstrated for the first time that applying butenafine in the infected skin of BALB/c mice decreased the size of the skin lesion as well as parasitism. Additionally, butenafine was formulated in cost-effective, easily scalable nanosystems prepared from generally regarded as safe excipients that were highly efficient at killing tissue amastigotes. These data provided preclinical proof of concept of the butenafine, administered in formulations or in the free form, which is effective in cutaneous leishmaniasis caused by *L. (L.) amazonensis*.

Physical data obtained demonstrated that both BUT-SNEEDS and BUT-SNEDDS gel have the potential to penetrate through the skin, since their particle sizes are 235 nm or below, with a low polydispersity index (<0.5) and a zeta potential around -24 mV. Previous studies showed that formulations containing particles with size lower than 300 nm, presenting low polydispersity (~ 0.4) and zeta potential below -25 mV, have high degree of stability, present low tendency to form aggregates, and have potential to penetrate through biological systems, such as the skin [42]. Thus, physical features suggested that both BUT-SNEEDS and BUT-SNEDDS gel are suitable formulations to be employed in studies aiming at analyzing butenafine efficacy by the topical route. In fact, studies employing Strat-M artificial membranes, that mimic the skin and transcutaneous permeation, showed that butenafine formulated into SNEDDS and SNEDDS gel presented high steady-state flux, permeability, and diffusion coefficients suggesting a faster permeability through the membrane than butenafine solubilized in PBS plus 1% DMSO; additionally, a lower lag time observed in both formulations showed that butenafine formulated into SNEDDS and SNEDDS gel diffused faster than butenafine through artificial membranes.

Healthy BALB/c mice were treated with BUT-SNEDDS and BUT-SNEDDS gel to analyze possible toxic effects of formulation in the skin of animals. In this case, no histological changes were observed in animals treated with butenafine, butenafine loaded in nanoenabled formulations, or blank formulations. Altogether, data suggested that free and nanoenabled formulations are not toxic to BALB/c skin after topical application for 15 consecutive days. In spite of that, it is important to point out that an inflammatory infiltrate was detected in the dermis of animals injected with Glucantime. The severe side effects induced by Glucantime can be avoided by applying it intralesionally; however, some patients can experience local inflammatory reactions, associated with the type IV hypersensitivity [43, 44], and a similar process may take place in BALB/c mice.

Butenafine, BUT-SNEDDS, and BUT-SNEDDS gel were able to decrease the size of the skin lesions in BALB/c mice. Blank formulations did not alter the course of the infection. BUT-SNEDDS gel was more efficient in reducing parasite load in the lesions compared to BUT-SNEDDS. According to data on artificial membrane permeation, both BUT-SNEDDS and BUT-SNEDDS gel have the same potential to permeate by membranes; however, BUT-SNEDDS has higher viscosity (data not shown), favoring the draining out of this formulation, that can alter the efficacy of this formulation. Moreover, animals treated with BUT-SNEDDS gel or butenafine demonstrated similar lesion size and tissue parasitism in comparison to the animals treated with Glucantime. In previous studies with terbinafine, the latter also was able to inhibit the development of the skin lesion in BALB/c mice infected with *L. (L.) major* [45], and humans naturally infected with *Leishmania* sp. receiving terbinafine by oral route or topically associated with Glucantime had improvements in the skin lesions [32, 33], suggesting that inhibitors of squalene epoxidase enzyme can be interesting targets to characterize new classes of leishmanicidal drugs. In addition to the therapeutic activity of butenafine, the topical route of application offers many advantages compared to injections, such as the possibility of self-administration, pain-free, no need for patient hospitalization, enable to bypass the liver metabolism of drugs, and more importantly is noninvasive; thus, this can be considered a useful nanomedicine to treat cutaneous leishmaniasis. By contrast, Glucantime, the first-line drug, although effective in the present study, for human treatment has been considered outdated, highly toxic, invasive, and painful, and more importantly, some patients are refractory to this treatment [46–48], which in fact can limit its efficacy.

The same pattern observed in the studies of the skin parasitism was found in the histological analysis. In the skin of the control groups [infected and nontreated (G1), infected and treated with blank SNEDDS (G2), or blank SNEDDS gels (G3)], an intense inflammatory infiltrate was observed and it was mainly composed by heavily infected macrophages. In the skin of animals treated topically with BUT-SNEDD (G4), intermediate number of amastigote forms was observed along with an intense inflammatory infiltrate composed by polymorphonuclear and mononuclear cells; in the skin of animals treated with BUT-SNEDD gel (G5), few amastigote forms were identified (inset in Figure 3(e)), and mononuclear cells were the main cells identified in the inflammatory infiltrate; additionally, fibroblasts were observed around the inflammatory cells that may be associated with the process of skin remodeling [49], suggesting a superior therapeutic activity of such formulation compared to BUT-SNEDD. A low number of amastigote forms inside big intracellular vacuoles from macrophages were observed in the skin of animals with butenafine (G6); furthermore, an inflammatory process composed by both mononuclear and polymorphonuclear cells was identified in focal areas of the skin. The features associated with the low number of parasitism along with focal areas suggested that butenafine was active in the experimental model cutaneous leishmaniasis. The skin of animals treated by intralesional route with Glucantime (G7) presented similar features than the skin from G4, since few amastigote forms were detected (inset in Figure 3(g)), but the inflammatory infiltrate persisted that can be an effect of low number of amastigote forms or even an effect of the drug, since Glucantime triggered an inflammatory response in the skin of healthy animals (Figure 1(g)) and humans [44].

In cutaneous leishmaniasis, IL-4 and IFN-*γ* cytokines play antagonistic roles, as IFN-*γ* is capable of activating macrophages that, in turn, will produce reactive species of nitrogen and oxygen and eliminate intracellular amastigote forms [50]. On the other hand, IL-4 aids CD4^+^ Th2 lymphocyte differentiation and inhibits Th1 generation [51]. In the present study, the level of IL-4 in treated animals was unaltered when compared to the control, suggesting differentiation of IL-4-producing cells stimulated by the parasite antigens. Conversely, high levels of IFN-*γ* were detected in animals treated with BUT-SNEDDS gel and butenafine, suggesting that butenafine has immunomodulatory activity, and at least partially, the leishmanicidal activity of this drug can be accounted due to its immunomodulation [52]. Surprisingly, cells from animals treated with SNEDDS did not change the profile of cytokine production. This can be explained by the low viscosity of SNEDDS compared to SNEDDS gels and the inability of SNEDDS to remain on the skin. On the other hand, mononuclear cells from animals treated with Glucantime produced low levels of both IL-4 and IFN-*γ* cytokines. Possibly, Glucantime eliminates high number of parasites quickly and the remaining ones are not able to induce the differentiation of specific Th1 or Th2 anti-*Leishmania* T lymphocyte clones. However, it was shown *in vitro* that butenafine, as well as other squalene epoxidase inhibitors [26, 34, 35], was able to eliminate amastigote forms after 24 h; thus, parasites can be eliminated slower, allowing antigens to circulate and maintaining clones of T cells. Recently, Yamamoto et al. [53] observed that cells from BALB/c mice infected with *L. (L.) amazonensis* and treated with amphotericin B also produced low amounts of IL-4 and IFN-*γ* cytokines, pointing out to the fact that low parasite numbers cannot stimulate a specific immune response, and in fact, a minimum level is needed to maintain an efficient inflammatory response. In addition, it was possible to observe that groups treated with BUT-SNEEDS gel or butenafine enhanced the efficacy of the immune response, since a strong Th1 immune response was triggered upon treatment, and as expected, the control groups produced high levels of IL-4 and developed an increased Th2 immune response.

In conclusion, butenafine chloride was successfully formulated as nanoenabled stable gels for topical administration. BUT-SNEDDS gel showed high flux across healthy mouse skin without causing any toxic, inflammatory, or allergic reactions. Additionally, infected BALB/c mice topically treated with BUT-SNEDDS gel or butenafine (vehicle) showed reduced lesion size and parasite load similar to that elicited by intralesional administration of Glucantime and these effects were associated with increase in IFN-*γ* levels. Taken together, transcutaneous drug delivery of butenafine can offer advantages over other invasive routes of administration currently in use towards a cost-effective, easily scalable, and safe topical repurposed therapy for leishmaniasis.

## Figures and Tables

**Table 1:** Mean particle size, polydispersity, and zeta potential of prepared batches of BUT-SNEDDS and BUT-SNEDDS gel (*n* = 4).

Formulation	Particle size (nm)	Polydispersity	Zeta potential (mV)
BUT-SNEDDS	185 ± 2	0.343 ± 0.024	−20.3 ± 3.0
BUT-SNEDDS gel	235 ± 11	0.458 ± 0.032	−24.3 ± 1.5
Blank SNEDDS	245 ± 27	0.578 ± 0.011	−14.8 ± 2.3
Blank SNEDDS gels	294 ± 85	0.605 ± 0.032	−22.6 ± 1.9

**Table 2:** Permeation parameters for butenafine and butenafine nanoenabled formulations across Strat membrane.

Parameter	BUT-SNEDDS gel	BUT-SNEDDS	Butenafine
Steady-state flux (*μ*g/cm^2^/h)	52.2 ± 2.7^∗^	51.29 ± 0.34^∗^	35.60 ± 12.10
Lag time (min)	2.14 ± 0.01^∗^	2.60 ± 0.01	5.28 ± 0.01
Permeability coefficient (cm^2^/h)	5.22 ± 0.27^∗^	5.13 ± 0.03^∗^	3.56 ± 1.21
Diffusion coefficient (cm/h)	0.57 ± 0.03^∗^	0.56 ± 0.01^∗^	0.39 ± 0.27

^∗^ *p* < 0.05 indicates statistical significance in comparison to the permeation parameters for butenafine.

## Data Availability

The data are available from the corresponding author upon request.
